# Supplementary material for: The Systems Biology Research Tool: evolvable open-source software
Source: BMC Syst Biol. 2008 Jun 29;2:55. doi: 10.1186/1752-0509-2-55 (PMC2446383; doi:10.1186/1752-0509-2-55)
Supplement: Additional file 1 — SBRT Archive. An archive of the current version of the Systems Biology Research Tool. [file 1752-0509-2-55-S1.zip › sbrt-1.4.0/doc/developers_guide/api/sbrt/shell/text/class-use/MapFormat.html]

Uses of Interface sbrt.shell.text.MapFormat


|  |  |  |
| --- | --- | --- |
| **Overview** \| **Package** \| **Class** \| **Use** \| **Tree** \| **Deprecated** \| **Index** \| **Help** | |  |
| PREV   NEXT | **FRAMES**    **NO FRAMES**     **All Classes** |  |


---


## **Uses of Interface sbrt.shell.text.MapFormat**

| Packages that use MapFormat | |
| --- | --- |
| **sbrt.shell.io** | Provides classes and interfaces for reading and writing files. |
| **sbrt.shell.io.fba** | Provides classes and interfaces for reading and writing files relevant to Flux Balance Analysis. |
| **sbrt.shell.mng.fba** | Provides classes and interfaces for creating Flux Balance Analysis process managers. |
| **sbrt.shell.mng.geom** | Provides classes and for creating geometry process managers. |
| **sbrt.shell.text** | Provides classes and interfaces for defining formats for basic objects. |
| **sbrt.shell.text.fba** | Provides classes and interfaces for defining formats for objects relevant to Flux Balance Analysis. |

Uses of MapFormat in sbrt.shell.io

| Methods in sbrt.shell.io that return MapFormat | |
| --- | --- |
| `MapFormat<K,V>` | `RomoFileSource.getLineFormat()`             Returns the map format used to create files of type `RomoFile`. |
| `MapFormat<K,V>` | `ComoFileSource.getLineFormat()`             Returns the map format used to create files of type `ComoFile`. |
| `MapFormat<K,V>` | `RomiFileSource.getLineFormat()`             Returns the map format used to create files of type `RomiFile`. |
| `MapFormat<K,V>` | `ComiFileSource.getLineFormat()`             Returns the map format used to create files of type `ComiFile`. |
| `MapFormat<K,V>` | `AbstractMapOutputFile.getMapFormat()`             Returns the format used to write lines to this file. |
| `MapFormat<K,V>` | `AbstractMapInputFile.getMapFormat()`             Returns the map format used to parse this file. |
| `MapFormat<K,V>` | `MapTextFile.getMapFormat()`             Returns the map format used by this file. |

Constructors in sbrt.shell.io with parameters of type MapFormat

`AbstractMapInputFile(java.lang.String fileName,
FileFormat fileFormat,
MapFormat<K,V> mapFormat)`
  
          Constructs a new map input file from the provided arguments.

`AbstractMapInputFile(java.lang.String fileName,
FileFormat fileFormat,
java.util.Set<? extends K> requiredKeys,
MapFormat<K,V> mapFormat)`
  
          Constructs a new map input file from the provided arguments.

`AbstractMapOutputFile(java.lang.String fileName,
FileFormat fileFormat,
MapFormat<K,V> mapFormat)`
  
          Constructs a new vector output file from the provided
arguments.

`AbstractMapOutputFile(java.lang.String fileName,
FileFormat fileFormat,
java.util.Set<? extends K> keys,
MapFormat<K,V> mapFormat)`
  
          Constructs a new map output file from the provided
arguments.

`AbstractMapOutputFile(java.lang.String fileName,
FileFormat fileFormat,
java.lang.String allVariables,
MapFormat<K,V> mapFormat)`
  
          Constructs a new map output file from the provided
arguments.

`ComiFile(java.lang.String fileName,
FileFormat fileFormat,
MapFormat<K,V> mapFormat)`
  
          Constructs a new row-oriented map input file from the
provided arguments.

`ComiFile(java.lang.String fileName,
FileFormat fileFormat,
java.util.Set<? extends K> requiredKeys,
MapFormat<K,V> mapFormat)`
  
          Constructs a new row-oriented map input file from the
provided arguments.

`ComiFile(java.lang.String fileName,
MapFormat<K,V> mapFormat)`
  
          Constructs a new row-oriented map input file from the
provided arguments.

`ComiFileSource(java.util.Iterator<java.lang.String> fileNameSource,
FileFormat fileFormat,
MapFormat<K,V> mapFormat)`
  
          Constructs a new source of column-oriented map input files.

`ComiFileSource(java.util.Iterator<java.lang.String> fileNameSource,
FileFormat fileFormat,
java.util.Set<? extends K> requiredKeys,
MapFormat<K,V> mapFormat)`
  
          Constructs a new source of column-oriented map input files.

`ComiFileSource(java.util.Iterator<java.lang.String> fileNameSource,
java.util.Set<? extends K> requiredKeys,
MapFormat<K,V> mapFormat)`
  
          Constructs a new source of column-oriented map input files.

`ComoFile(java.lang.String fileName,
FileFormat fileFormat,
java.util.Set<? extends K> columnHeaderVariables,
MapFormat<K,V> vectorFormat)`
  
          Constructs a new column-oriented map output file
from the provided arguments.

`ComoFile(java.lang.String fileName,
FileFormat fileFormat,
java.lang.String allColumnHeaderVariables,
MapFormat<K,V> vectorFormat)`
  
          Constructs a new column-oriented vector output file
from the provided arguments.

`ComoFile(java.lang.String fileName,
java.util.Set<? extends K> columnHeaderVariables,
MapFormat<K,V> vectorFormat)`
  
          Constructs a new text-based column-oriented map output file
from the provided arguments.

`ComoFileSource(java.util.Iterator<java.lang.String> fileNameSource,
FileFormat fileFormat,
MapFormat<K,V> mapFormat)`
  
          Constructs a new source of column-oriented map output files.

`ComoFileSource(java.util.Iterator<java.lang.String> fileNameSource,
FileFormat fileFormat,
java.util.Set<? extends K> keys,
MapFormat<K,V> mapFormat)`
  
          Constructs a new source of column-oriented map output files.

`ComoFileSource(java.util.Iterator<java.lang.String> fileNameSource,
FileFormat fileFormat,
java.lang.String keys,
MapFormat<K,V> mapFormat)`
  
          Constructs a new source of column-oriented map output files.

`RomiFile(java.lang.String fileName,
FileFormat fileFormat,
MapFormat<K,V> mapFormat)`
  
          Constructs a new row-oriented map input file from the provided arguments.

`RomiFile(java.lang.String fileName,
FileFormat fileFormat,
java.util.Set<? extends K> requiredKeys,
MapFormat<K,V> mapFormat)`
  
          Constructs a new row-oriented map input file from the provided arguments.

`RomiFile(java.lang.String fileName,
MapFormat<K,V> mapFormat)`
  
          Constructs a new row-oriented map input file from the provided arguments.

`RomiFile(java.lang.String fileName,
java.util.Set<? extends K> requiredKeys,
MapFormat<K,V> mapFormat)`
  
          Constructs a new row-oriented map input file from the provided arguments.

`RomiFileSource(java.util.Iterator<java.lang.String> fileNameSource,
FileFormat fileFormat,
MapFormat<K,V> mapFormat)`
  
          Constructs a new source of row-oriented map input files.

`RomiFileSource(java.util.Iterator<java.lang.String> fileNameSource,
FileFormat fileFormat,
java.util.Set<? extends K> requiredKeys,
MapFormat<K,V> mapFormat)`
  
          Constructs a new source of row-oriented map input files.

`RomiFileSource(java.util.Iterator<java.lang.String> fileNameSource,
MapFormat<K,V> mapFormat)`
  
          Constructs a new source of row-oriented map input files.

`RomoFile(java.lang.String fileName,
FileFormat fileFormat,
MapFormat<K,V> vectorFormat)`
  
          Constructs a new row-oriented vector output file from the
provided arguments.

`RomoFile(java.lang.String fileName,
FileFormat fileFormat,
java.util.Set<? extends K> rowHeaderVariables,
MapFormat<K,V> mapFormat)`
  
          Constructs a new row-oriented map output file
from the provided arguments.

`RomoFile(java.lang.String fileName,
FileFormat fileFormat,
java.lang.String allRowHeaderVariables,
MapFormat<K,V> vectorFormat)`
  
          Constructs a new row-oriented vector output file
from the provided arguments.

`RomoFile(java.lang.String fileName,
MapFormat<K,V> vectorFormat)`
  
          Constructs a new plain text, row-oriented vector output
file from the provided arguments.

`RomoFileSource(java.util.Iterator<java.lang.String> fileNameSource,
FileFormat fileFormat,
MapFormat<K,V> mapFormat)`
  
          Constructs a new source of row-oriented map output files.

`RomoFileSource(java.util.Iterator<java.lang.String> fileNameSource,
FileFormat fileFormat,
java.util.Set<? extends K> keys,
MapFormat<K,V> mapFormat)`
  
          Constructs a new source of row-oriented map output files.

`RomoFileSource(java.util.Iterator<java.lang.String> fileNameSource,
FileFormat fileFormat,
java.lang.String keys,
MapFormat<K,V> mapFormat)`
  
          Constructs a new source of row-oriented map output files.

Uses of MapFormat in sbrt.shell.io.fba

| Methods in sbrt.shell.io.fba that return MapFormat | |
| --- | --- |
| `MapFormat<java.lang.String,java.util.Set<LinearComb<java.lang.String>>>` | `FluxCapInputFile.getMapFormat()`             Returns the map format used to parse this file. |

Constructors in sbrt.shell.io.fba with parameters of type MapFormat

`FluxIntervalInputFile(java.lang.String fileName,
FileFormat fileFormat,
java.util.Set<LinearComb<java.lang.String>> requiredVariables,
MapFormat<LinearComb<java.lang.String>,Interval> mapFormat)`
  
          Constructs a new flux-interval input file.

Uses of MapFormat in sbrt.shell.mng.fba

| Methods in sbrt.shell.mng.fba with parameters of type MapFormat | |
| --- | --- |
| `static FluxIntervalInputFile` | `IntervalVariationManager.getIntervalInputFile(java.lang.String fileName, Fluxome fluxome, MapFormat<LinearComb<java.lang.String>,Interval> mapFormat)`             Returns the flux interval input file with the provided name. |

Uses of MapFormat in sbrt.shell.mng.geom

| Methods in sbrt.shell.mng.geom with parameters of type MapFormat | |
| --- | --- |
| `static java.util.Map<java.lang.String,java.lang.Double>` | `CdHarManager.getInitialPoint(java.lang.String fileName, MapFormat<java.lang.String,java.lang.Double> format, java.util.Set<java.lang.String> variables)`             Opens the file with the provided name and return the vector it contains. |

Uses of MapFormat in sbrt.shell.text

| Subinterfaces of MapFormat in sbrt.shell.text | |
| --- | --- |
| `interface` | `AppInputFileLineFormat`             This interface is used to represent the format of lines in process input files. |

| Classes in sbrt.shell.text that implement MapFormat | |
| --- | --- |
| `class` | `AbstractMapFormat<K,V>`             This abstract class is a skeleton implementation of `MapFormat`. |
| `class` | `AppInputFileLineFormatVersion1`             This class is a concrete implementation of `AppInputFileLineFormat`. |
| `class` | `ColonMapFormatV1<K,V>`             This class is a concrete implementation of `MapFormat`. |
| `class` | `EqualsMapFormatV1<K,V>`             This class is a concrete implementation of `MapFormat`. |

Uses of MapFormat in sbrt.shell.text.fba

| Subinterfaces of MapFormat in sbrt.shell.text.fba | |
| --- | --- |
| `interface` | `ConstraintsFileLineFormat<E>`             This interface is used to represent formats for flux constraints files. |
| `interface` | `FbaOptFileLineFormat`             This interface is used to represent formats of FBA optimization file lines. |
| `interface` | `FluxCapFormat`             This interface is used to represent the formats of lines of flux cap-containing files. |
| `interface` | `FluxomeSolutionFileLineFormat`             This interface is used to represent formats for solutions to the linear system of equations formed by a fluxome. |
| `interface` | `FluxVectorFormat`             This interface is used to represent formats for flux vectors. |

| Classes in sbrt.shell.text.fba that implement MapFormat | |
| --- | --- |
| `class` | `ConstraintsFileLineFormatV1`             This class is a concrete implemenation of `ConstraintsFileLineFormat`. |
| `class` | `ConstraintsFileLineFormatV2`             This class is a concrete implemenation of `ConstraintsFileLineFormat`. |
| `class` | `FbaOptFileLineFormatV1`             This class is a concrete implementation of `FbaOptFileLineFormat`. |
| `class` | `FluxCapFormatV1`             This class is a concrete implementation of `FluxCapFormat`. |
| `class` | `FluxomeSolFileLineFormatV1`             This class is a concrete implemenation of `FluxomeSolutionFileLineFormat`. |
| `class` | `FluxVectorFormatV1`             This class is a concrete implemenation of `FluxVectorFormat`. |
| `class` | `IrfFormatV1`             This class is used to format irreversible reaction fluxome files. |

---


|  |  |  |
| --- | --- | --- |
| **Overview** \| **Package** \| **Class** \| **Use** \| **Tree** \| **Deprecated** \| **Index** \| **Help** | |  |
| PREV   NEXT | **FRAMES**    **NO FRAMES**     **All Classes** |  |


---
